# Supplementary material for: Social support for breast cancer patients in the occupied Palestinian territory
Source: PLoS One. 2021 Jun 18;16(6):e0252608. doi: 10.1371/journal.pone.0252608 (PMC8213135; doi:10.1371/journal.pone.0252608)
Supplement: Attachment — Submitted filename: Response to reviewers.docx [file pone.0252608.s001.docx]

**Response to Reviewers**

*In what follows we outline the main changes that we have made to our manuscript by answering to each of the reviewers’ requests.*

*As suggested, we have tracked the changes made to our original submission. We provide also another file that shows the changes made by an English proofreader. We also provide a final, clean version.*

Reviewer #1: This is an excellent article!

It throws light on a Palestinian population living under occupation and difficult conditions. It gives information about how women cope with breast cancer under such circumstances, but also gives information about of how women in general cope with breast cancer. The results are therefore relevant also for other populations. - The analysis is well done, and the background (theory and previous literature) is relevant.

Regarding question 2 (above): (Statistical analysis), I answered "yes" as the qualitative analysis is good. There are, of course, no statistical analyses in a qualitative investigation.

Regarding question 3 (above): (Whether all data are available), - I also answered "yes", as all relevant data seem to be analysed and sufficiently reported. Needless to say: one cannot, and should not, for ethical reasons, report absolutely all data in a qualitative report.

I suggest only these corrections: On page 17 a name, "Nadia" is mentioned. That should be omitted, and only the initial "N" should be used (as "N" is used for this informant a few sentences earlier in the manuscript. - In another place "Su" appears as what looks like a name. - That too should perhaps be shortened to just "S".

*Changed as suggested.*

Reviewer #2: The article covers an important topic, however the paper needs considerable revision to be of publishable quality. One of the main (major) issues with the paper is that the authors do not adequately engage with the data presented through quotes; some statements are made without being adequately supported or explained. They may be true, but need more explanation and evidence from the authors, for example the issue of breast cancer being stigmatized. There also needs to be more thorough engagement with the literature in the discussion section. The authors compare the findings about husband support with others in the Arab world, but in their discussion there is an implicit assumption that 'Arab' culture is homogeneous, which is not the case, and cultures within any place are also not static.

More specific comments:
1) the transition from the introduction to the objectives could be improved by making the case as to why this study is important in Palestinian context
2) objectives should be in introduction rather than materials and methods; objectives also need to be written more clearly, especially first line
3) line 141 part about literature that 'gave voice' is a bit of an odd statement and perhaps better to say what methods they drew on

*1-2-3: Objectives have been moved in the introduction and partially reformulated. In order to making a case for why the study is important, we added few lines about cancer incidence in the Palestinian territory and the relevance of studying coping strategies under these local difficult circumstances (being under occupation) . In what used to be line 141 “gave voice” has been erased and the line rephrased.*

*Reference to the importance of local culture (addressed by Rev2 at the beginning of this letter) and culturally sensitive studies has been added at the end of the introduction.*

4) line 148, incorrect statement about Beit Jala, it is one of the major government hospitals in the West Bank providing oncology services but not the only one

*Correction made*

5) line 158, not clear what is meant by security and travel as the justification. Is this for the researchers coming from abroad and limitations on their stay? Better to clarify

*Clarification added*

6) justification needs to be provided for inclusion criteria, especially 3 and 4. In local context, verbal consent is often deemed appropriate by IRB and ethical review committees, why did researchers insist on signed consent?

*Few lines of explanation have been added. Verbal consent was asked and followed by a written consent that also provided the opportunity to give the patients written information about the research and researchers’ contacts*

7) line 167 needs to be explained further

*This line has been eliminated*

8) line 180-'adherence to research' odd wording

*Rephrased*

9) line 193, what was rendered sufficient?

*Clarification added*

10) line 215, not clear if according to literature or the women interviewed

*Clarification added*

11) line 227-228 where authors state 'attributed to young age and marital status"- it is not clear what is meant and this should be explained more, examples from the data would also be useful.

*This statement has been rephrased and moved later on in the discussion section.*

12) line 229, the statement makes it seem like all women stated they had no support from those outside family, but quotes later on contradict this (e.g. extracts 8 and 9)

*Rephrased*

13) for discussion of woman whose husband abandoned her, the quote on line 355 also states he comes once a month. It is a bit confusing.

*The interviewee states clearly that their marriage is finished. She still meets the husband occasionally but without having the feeling of a real communication between them*

14) beginning line 358: authors jump to loss of work, the transition is abrupt

*A line that reconnects with the financial support topic has been added*

15) paragraph beginning line 369, authors use N. and then Nadia, better to stick to one or the other and maybe initial for consistency, and ethical/privacy considerations.

*Changed in N.*

16) same participant as above, authors state she didn't tell her family and then talk about support from friends. Were there family members who knew about her diagnosis and not support her? it is not clear

*Rephrased. (She did not tell her diagnosis to the family).*

17) statement beginning on line 396 about family playing key role. This may be true, but authors should support through examples from study or references in the literature.

*The statement has been moved after extracts 18,19,20 that provide evidence for it*

18) lines 437-439, authors talk about lack of reliable information. From whom? The internet or is this more specific to Palestinian context? It reads as if it is more specific to Palestinian context, but at the same time it is not really explained. The references to unreliable information in Arabic on the internet would not limit this issue to Palestinian context.

*Specification added*

19) line 442, what negative impact? Explain.

*Few lines of explanation have been added*

20) line 468, not clear how explanation going back to political context explains centrality of husband as support provider. It is not clear and not convincing from the argument. Also, why should we assume that husbands won't be supportive?

*Explanation added (socio-political restriction to mobility obstruct access to both the health care system and the social support networks such as the extended family and other members of the nuclear family). A previous paragraph of the discussion section describes how in the literature on Arab countries the role of the husband as a companion and caregiver of women with breast cancer is controversial, picturing often the husband as a non supporting source.*

21) statement that breast cancer is stigmatized needs to be explained further.

*Main results of the studies quoted on this topic are reported.*

22) for ethical considerations, were patients also assured that their participation (or not) would not affect their access to treatment?

*Yes. Moreover, several patients already completed treatments.*

23) Authors used an inductive approach, did this impact what kinds of support were identified? Were there other forms of support that were not identified during this approach? Important to discuss limitations of analytical approach as well

*Limitations and reference to other analytical approaches have been added*

24) the manuscript can use some copy editing, some use of language reads as a bit odd.

*The manuscript has been proofread by a fluent English speaker and changes have been made.*
