# Supplementary material for: Social support for breast cancer patients in the occupied Palestinian territory
Source: PLoS One. 2021 Jun 18;16(6):e0252608. doi: 10.1371/journal.pone.0252608 (PMC8213135; doi:10.1371/journal.pone.0252608)
Supplement: Attachment — Submitted filename: response to reviewers.docx [file pone.0252608.s002.docx]

Dear PLOS ONE Academic Editor and anonymous reviewers,

My co-authors and I are grateful for the attention given to our manuscript PONE-D-20-3697R1

and the suggestions offered by the Academic editor. We changed the manuscript accordingly in lines 478-481 and rephrase the reference to western individualistic culture as recommended.

Kind regards,

The Authors
